# Observation of electron orbital signatures of single atoms within metal-phthalocyanines using atomic force microscopy

**DOI:** 10.1038/s41467-023-37023-9

**Published:** 2023-03-16

**Authors:** Pengcheng Chen, Dingxin Fan, Annabella Selloni, Emily A. Carter, Craig B. Arnold, Yunlong Zhang, Adam S. Gross, James R. Chelikowsky, Nan Yao

**Affiliations:** 1 Princeton Materials Institute, Princeton University, Princeton, NJ 08540-8211 USA; 2 McKetta Department of Chemical Engineering, University of Texas at Austin, Austin, TX 78712-1589 USA; 3 Department of Chemistry, Princeton University, Princeton, NJ 08544-0001 USA; 4 Department of Mechanical and Aerospace Engineering and the Andlinger Center for Energy and the Environment, Princeton University, Princeton, NJ 08544-5263 USA; 5 Princeton Plasma Physics Laboratory, Princeton, NJ 08540-6655 USA; 6 ExxonMobil Technology and Engineering Company, Annandale, NJ 08801-3096 USA; 7 Department of Physics, University of Texas at Austin, Austin, TX 78712-1192 USA; 8 Center for Computational Materials, Oden Institute for Computational Engineering and Sciences, University of Texas at Austin, Austin, TX 78712-1229 USA

**Keywords:** Electronic properties and materials, Two-dimensional materials, Scanning probe microscopy

## Abstract

Resolving the electronic structure of a single atom within a molecule is of fundamental importance for understanding and predicting chemical and physical properties of functional molecules such as molecular catalysts. However, the observation of the orbital signature of an individual atom is challenging. We report here the direct identification of two adjacent transition-metal atoms, Fe and Co, within phthalocyanine molecules using high-resolution noncontact atomic force microscopy (HR-AFM). HR-AFM imaging reveals that the Co atom is brighter and presents four distinct lobes on the horizontal plane whereas the Fe atom displays a “square” morphology. Pico-force spectroscopy measurements show a larger repulsion force of about 5 pN on the tip exerted by Co in comparison to Fe. Our combined experimental and theoretical results demonstrate that both the distinguishable features in AFM images and the variation in the measured forces arise from Co’s higher electron orbital occupation above the molecular plane. The ability to directly observe orbital signatures using HR-AFM should provide a promising approach to characterizing the electronic structure of an individual atom in a molecular species and to understand mechanisms of certain chemical reactions.

## Abstract (web summary)

Resolving the orbital structure of single atoms is challenging and of great importance for understanding basic chemistry. Here, the authors demonstrate that the orbital occupation difference of single Fe/Co atoms within molecules can be distinguished with high resolution AFM imaging and spectroscopy.

## Introduction

Real-space experimental observation of localized electron orbital signatures for individual atoms within complex systems can elucidate how atoms interact with each other and provide critical information on the dissociation and formation of chemical bonds needed for identifying reaction pathways. However, the direct measurement of the electronic structure of a single atom or a chemical bond is challenging. Several experimental methods have enabled probing of molecular orbital distributions under certain conditions, including angle-resolved photoemission spectroscopy^1,2^, high harmonic interferometry^3^, and photoionization microscopy^4^. In real space, orbital-related information can be obtained with scanning tunneling microscopy (STM)^5–10^, which images the spatially resolved local density of states near the Fermi level^11^. In addition, HR-AFM with molecularly functionalized tips has been used for quantitative structural measurements on organic molecules with spectacular atomic resolution^12,13^. Bond order^14,15^ and heteroatom^16,17^ discrimination, and even real-space imaging of individual atoms^18,19^ and intermolecular bonds have been reported^20,21^. These experimental advances have been accompanied by the innovation of new algorithms and an exponential increase of computer processing power, which provides an avenue for solutions of the electronic structure of complicated molecular systems using density functional theory (DFT)^22,23^-based methods. These solutions offer accurate simulations of atomic force imaging and the possibility of utilizing HR-AFM to directly probe the electronic structure of atoms at the orbital level.

Here, we center on single transition-metal atoms, Fe and Co, within Fe-phthalocyanine (FePc) and Co-phthalocyanine (CoPc) on Cu(111) surfaces using a qPlus type nc-AFM with a CO-functionalized tip. In constant-height AFM images, the Co atom appears brighter with four distinct lobes on the horizontal plane while the Fe appears to have a nearly “square” shape. In pico-force spectroscopy measurements, distinct force-distance curves and a difference of about 5 pN at the minimum point were observed on the Fe and Co centers of FePc and CoPc. These differences can be ascribed to the different ${d}_{{xz}}$, ${d}_{{yz}}$and ${d}_{{z}^{2}}$ orbital occupations of the Fe and Co centers. Our DFT-based AFM simulations further show that for both Fe and Co, changes in the occupation of the ${d}_{{z}^{2}}$ orbitals lead to different images and interaction forces in AFM measurements. Our results demonstrate that electronic orbital signatures can be revealed by real-space HR-AFM imaging and spectroscopy.

## Results and discussion

### STM topography of Fe/CoPc on Cu(111) surface

**Fig. 1:**
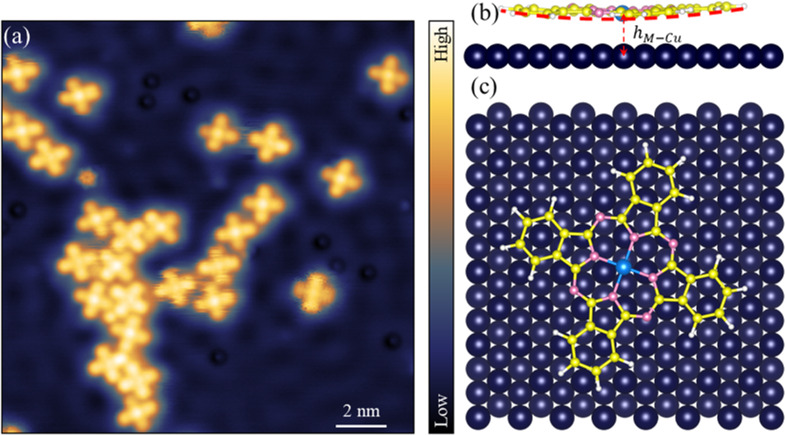
STM topography and schematic views of the adsorption geometry. **a** Low-magnification STM image of FePc and CoPc molecules using a CO tip (*V* = 100 mV, *I* = 30 pA). Schematic side (**b**) and top (**c**) views of the relaxed FePc molecule adsorbed on a Cu(111) substrate. The red dashed curve in **b** indicates the calculated bent adsorption geometry. ${h}_{M-{Cu}}$ is the MPc adsorption height and is defined as the distance between the metal atom within the Pc and the surface of the Cu substrate. ${h}_{{Fe}-{Cu}}$ = 261.4 pm, ${h}_{{Co}-{Cu}}$ = 248.8 pm. Blue: Fe, yellow: C, pink: N, white: H, dark purple: Cu.

A large-scale STM topographic image of the sample surface shows the overall metal phthalocyanine (MPc) distribution on a Cu(111) substrate after depositing FePc and CoPc molecules sequentially (Fig. 1a). Both MPcs have a coverage of less than 10% of one monolayer. The cross-shape of FePc and CoPc molecules are clearly evident. Figure 1b, c shows schematic side and top views of the relaxed FePc molecule adsorbed on a Cu(111) substrate where the Fe atom is at a bridge site^24^.

### HR-AFM characterization and analysis

**Fig. 2:**
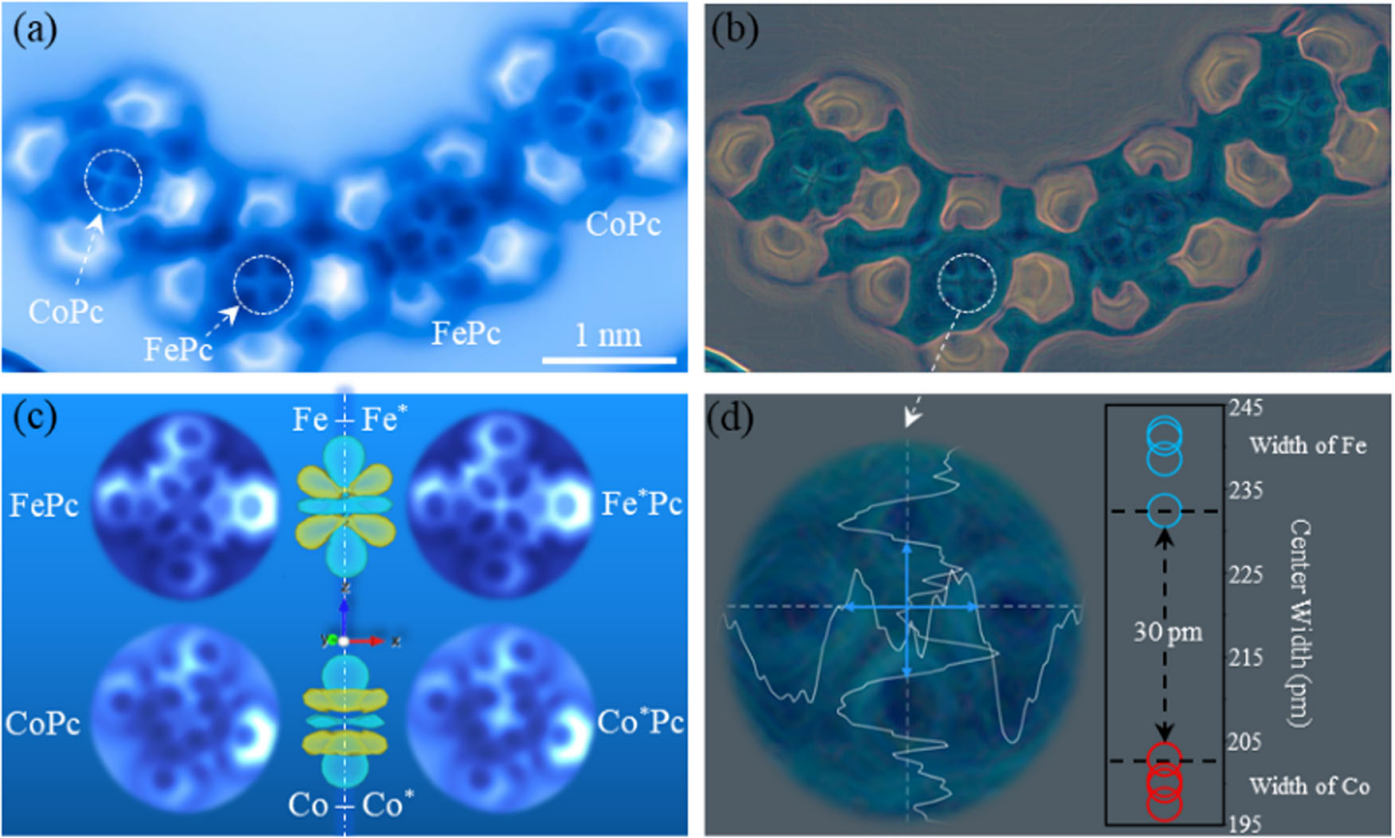
AFM images of FePc and CoPc on a Cu(111) surface. **a** Experimental constant-height AFM frequency-shift images (*V* = 0 V, tip amplitude = 100 pm) using a CO tip at a tip height of −10 pm with respect to our 100 mV/100 pA STM set point. The two white dashed circles highlight the main differences between these two molecules—the central metal atom. **b** Glow-edges filtered experimental AFM image (based on **a**). **c** Simulated AFM images with a CO tip at a tip height of −10 pm (see Supplementary Information for the definition of tip height in simulation). Left panel: spin-polarized DFT calculations; right panel: spin-paired DFT calculations (indicated by a superscript *). On the midline, the orbital-like figures are the calculated total electron density differences between MPc and M*Pc (MPc–M*Pc). Yellow: positive, cyan: negative. Isovalue: 0.003 e^–^/bohr^3^. **d** Estimated width (in pm) of the central part of the MPcs based on the signal strength—*I* value. The white dashed arrow pointing from **b** to **d** indicates a zoomed-in image of the central part of the left FePc molecule. The white curves are calculated *I* values along the corresponding dashed axes. The blue arrows illustrate how we define the width of the square based on *I* values. Top panel: FePc (in blue), bottom panel: CoPc (in red). Each MPc has two widths and corresponds to two circles. The gap between the two dashed black lines (the highest red and lowest blue circles) shows a minimum difference of 30 pm.

In Fig. 2a, we present a HR-AFM image showing the sub-molecular structure of the FePc and CoPc molecules. The image was taken using a CO-functionalized tip operated in a constant-height scanning mode (see Supplementary Fig. 2 for images taken at larger tip heights and the corresponding DFT-calculated 3D electron density maps). For both FePc and CoPc, the internal features of the carbon heterocyclic skeleton can be resolved, as well as the central metal atoms. For both MPc molecules, the peripheral carbon rings are slightly brighter than the internal carbon-carbon bonds. This indicates that the molecular plane bends upward^25^ as illustrated by the red dashed curve in our calculated structure (Fig. 1b). We find that, FePc and CoPc can be distinguished by comparing details in the metal centers, as highlighted by two white dashed circles for the pair on the left: (1) Co appears brighter than Fe; (2) Co shows a more pronounced extension of the four lobes along the Co-N bonds while the Fe atom displays a more square-like shape with a wider dimension. Similar features are also observed for the FePc and CoPc pair on the right. We apply a glow-edges filter to these MPcs to enhance the contrast of these features (Fig. 2b).

We propose that the distinct features of the center metal atoms originate from the different electron occupations within the $3d$-orbital manifold. To confirm this hypothesis, we compare simulated AFM images obtained using (1) spin-polarized DFT calculations (with spin states optimized before AFM simulations) for FePc and CoPc molecules (Fig. 2c—left panel) and (2) spin-paired DFT calculations (net magnetic moment = 0) for fictitious diamagnetic Fe^*^Pc and Co^*^Pc (Fig. 2c—right panel). We also display the computed total electron density differences between MPc and M^*^Pc (${\rho }_{{{{{{{\rm{spin}}}}}}}-{{{{{{\rm{polarized}}}}}}}}\left({{{{{\rm{MPc}}}}}}\right)-{\rho }_{{{{{{{\rm{spin}}}}}}}-{{{{{{\rm{paired}}}}}}}}\left({{{{{{\rm{M}}}}}}}^{*}{{{{{\rm{Pc}}}}}}\right)$, Fig. 2c—middle panel). For both molecules, we find a region where the M^*^Pcs have a higher electron density (in cyan) originating from the out-of-plane orbital(s) along the surface normal. As a result, the metal centers in the simulated AFM images for M^*^Pcs appear brighter and smaller. This simulation agrees with our suggestions, in terms of (1) brightness and (2) shape, that differences in nc-AFM images of FePc and CoPc come from different electron occupations of their orbitals. We further estimate the widths of the center parts of the MPc molecules based on the luminance signal strength (*I* value), using the filtered/enhanced AFM image (Fig. 2b) (see Eq. (1) in the “Experimental methods” section for how we calculate the *I* value). In Fig. 2d, the apparent width of the Fe center (blue circles) is at least 30 pm broader than Co (red circles), which corresponds to about a 15% difference despite Co having one more electron. This may be due to the fact that (1) Co has a larger screened nuclear charge that shrinks its 3d orbitals more than Fe, and/or (2) Fe has a larger in-plane and a smaller out-of-plane electron occupancy. Distinguishing atoms that only differ by one nuclear charge using AFM with an inert tip, which only measures the subtle electron density distributions instead of interacting with the specimen chemically^26–28^, is extremely challenging. For example, even an HR-AFM (CO tip) can barely distinguish N from C atoms unless a specific treatment is performed^16^. Subatomic structures of single adatoms and small clusters were resolved using HR-AFM^18^. The toroidal symmetry contrast of Cu/Fe adatom was attributed to electrostatic attraction at the center and Pauli repulsion at the circumference. Here, we propose that the 15% difference in our measurement is more likely to originate from the different electron occupancy of Fe’s and Co’s $3d$ orbitals.

### Force spectroscopy measurements

**Fig. 3:**
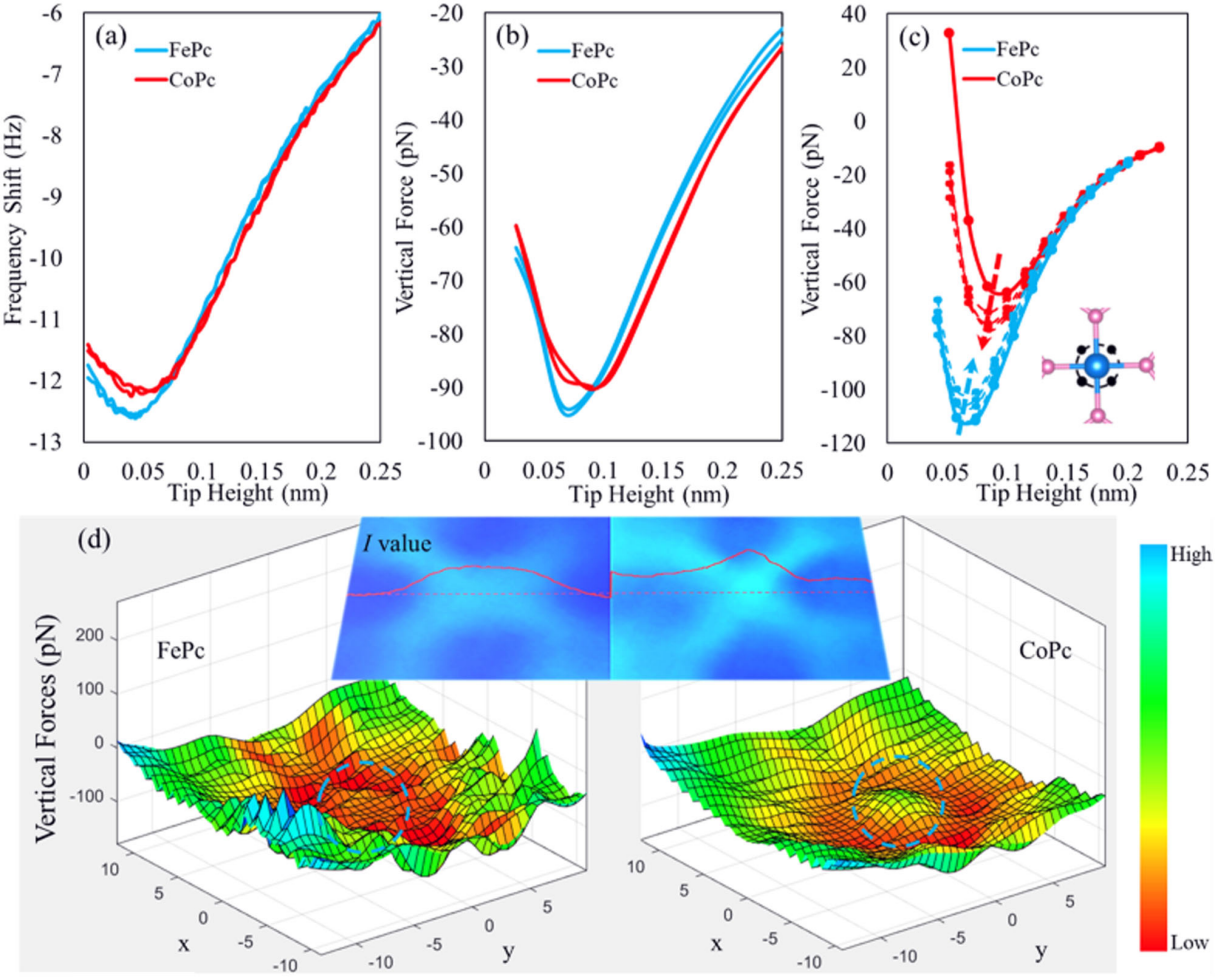
Measured and computed forces. **a** Measured frequency shift (Hz) and **b** vertical force (pN) acting on the CO tip when it is placed on the top of a center metal atom. **c** Spin-polarized DFT-predicted vertical forces (pN) acting on the CO tip. In **c**, the solid curves correspond to a configuration where the tip is directly on top of the metal atom. The dashed curves correspond to configurations where the tip is horizontally shifted away from the center by ~60 pm as indicated by the four black dots (in the inset) surrounding the metal atom. The dashed arrows indicate the trend of change in forces when the tip was displaced away from the center. **d** Side views of the calculated tip-MPc interaction force distribution. The inset shows the zoomed-in version of the center part (circled in blue on the force surfaces) of two MPcs (left: FePc, right: CoPc) in the AFM image of Fig. 2a. The red curve shows the calculated *I* values along the horizontal red dashed line in the middle. The units for the *x* and *y* axes are bohr.

$\varDelta f(z)$ force spectrum measurements were performed on top of the Fe and Co atoms. As shown in Fig. 3a, the frequency shift ($\varDelta f$) spectra obtained on four individual metal atoms (Fig. 2a) from molecule #1 (left) to molecule #4 (right) are well separated into two groups: the spectra from molecules #1 and #4 (red—Co), and from molecules #2 and #3 (blue—Fe). At the minimum points of the spectra, Fe and Co have a frequency shift difference of about 500 mHz, which is well above the system noise level (less than 100 mHz) using the same system parameter settings. The $\varDelta f(z)$ curves are converted to force-distance curves^29^ in Fig. 3b. Fe and Co atoms can be distinguished from both the frequency-shift and force spectra. At large tip-sample separations, Co exerts a larger attractive force on the tip. When the tip gets closer to the sample, a repulsive component to the interaction between the metal center and tip emerges, decreasing the force (that remains net attractive). The force curves cross at a turning point of about 90 pN. Figure 3b shows that by comparing the locations of the respective force curve minima, it is evident that the vertical interaction force on Co is about 5 pN less attractive than on Fe. Using the same tip, the force difference is clearly seen for adjacent FePc and CoPc in similar imaging environments. It should be noted that AFM measured interaction forces can be strongly tip dependent. Our measurements are performed with the same tip on neighboring molecules to exclude any perturbations from tip differences (also see Supplementary Fig. 7) on the metal base for CO adsorption and surface imperfection. The force spectra computed with spin-polarized DFT (Fig. 3c, solid curves) are in good qualitative agreement with the experimental force curves, with Co exhibiting a less attractive force than Fe at their respective minima. However, the calculated force difference between the curves for Fe and Co is larger than the measured value of 5 pN. A possible explanation is that in the experiment, the force curves were not measured exactly above the metal centers whereas in the calculations, we ignored this uncertainty and placed the tip directly above the metal centers.

To validate this hypothesis and determine how the force changes as a function of tip position, we performed a simulation wherein the tip is displaced from the central metal atom by ~ 60 pm. This displacement is slightly less than one third of the metal-N bond length, horizontally (i.e., in the x–y plane), as illustrated by the black dots in the inset of Fig. 3c. The dashed arrows indicate how the force changes as the tip is moved away from the center, which reduces the difference between the computed forces for Fe and Co. The trend upon moving away from on top of the metal atom also indicates higher occupation of out-of-(surface) plane d-orbitals for Co. The force becomes indeed more attractive upon this displacement. This trend is consistent with what one would expect from decreased Pauli repulsion by moving away from the Co center. The opposite trend is predicted for Fe, indicating lower occupation of such out-of-plane orbitals and larger occupation of in-plane orbitals. In other words, our calculations show a higher Pauli repulsion force at Fe’s circumference compared with its center. 3D tip–MPc force distributions also were computed to visualize the intermolecular interactions above the metal centers (indicated by the blue-dash circles in Fig. 3d). The gradient (in the $z$-direction) of the tip-sample interaction force is proportional to the frequency shift, which is a direct representation of the AFM image. We find that the force surface around the Fe atom is relatively flat, suggesting main contributions from the ${d}_{{xy}}$ and ${d}_{{x}^{2}-{y}^{2}}$ orbitals. In contrast, the force surface around the Co exhibits an undulation that is likely related to the $z$-oriented orbitals (${d}_{{xz}}$, ${d}_{{yz}}$ and ${d}_{{z}^{2}}$). This $z$ contribution can explain why a less attractive force was measured above the Co atom. As an additional verification, we display experimental AFM images of FePc and CoPc side by side and compute the *I* values (red curve in the inset of Fig. 3d) across the metal center. The shape of the curve above the metal atom center agrees well with the DFT-derived force distribution, confirming that different orbital contributions can be distinguished using both force spectra and HR-AFM images with a CO-functionalized tip.

### Orbital signature analysis

**Fig. 4:**
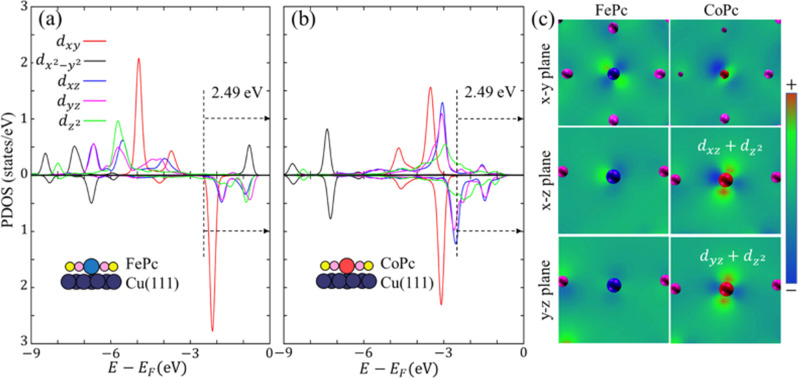
PDOS around the metal centers within MPcs. DFT-computed PDOS around Fe (**a**) and Co (**b**) atoms with MPcs on Cu(111) surfaces. The top and bottom panels correspond to spin-up and spin-down states, respectively. The CO tip is excluded in these results. **c** 2D volume slice views of the combined states in an energy window of 2.49 eV below the Fermi level as indicated by the black dashed line and arrows in **a** and **b**. In the x–y plane, Co appears smaller because its computed adsorption height is about 13 pm smaller than that of Fe (i.e., it is closer to the Cu surface). The spheres correspond to atoms colored as follows, blue: Fe, red: Co, pink: N, yellow: C, dark purple: Cu.

To obtain further insight into the local electronic structure of the metal atoms, we used spin-polarized pseudopotential DFT to compute the local magnetic moment (LMM) and the projected density of states (PDOS) around the center metal atoms within the MPcs (the CO tip is excluded). The calculated LMM of Fe (2.34 µ_B_) is significantly larger than that of Co (0.49 µ_B_), indicating a larger number of unpaired electrons on the Fe atom. Our observation explains why the AFM image of FePc is notably different from Fe*Pc in terms of both brightness and shape of the central part, while CoPc and Co*Pc have a different brightness but appear similar in shape (Fig. 2c). From the PDOS analysis, we find that both Fe and Co have large contributions from ${d}_{{xy}}$ and ${d}_{{x}^{2}-{y}^{2}}$ orbitals in both spin manifolds (indicating these two in-plane orbitals are doubly occupied in both MPcs) while Co has larger contributions from ${d}_{{z}^{2}}$, ${d}_{{xz}}$ and ${d}_{{yz}}$ orbitals near the Fermi level (Fig. 4a, b). The PDOS and LMMs are consistent with Fe being intermediate-spin *d*^6^ Fe(II), with two singly occupied out-of-plane orbitals while Co is low-spin *d*^7^ Co(II), with its three other electrons occupying just two of the three out-of-plane orbitals. These calculated PDOS deviate from the gas-phase ones^30^ due to strong coupling with the substrate. Both MPcs gain electronic charge(s)^31^ from the substrate which induces a charge redistribution within the metal atoms^32^. In addition, the computed adsorption height, which is defined as the distance between Co/Fe atoms and the Cu substrate, of CoPc (248.8 pm) is about 13 pm lower than that of FePc (261.4 pm). In order to confirm that the adsorption height only plays a negligible role in the observed features of Co and Fe under our experimental conditions, we intentionally lifted the CoPc complex upward so that we could compare the Co and Fe atoms in the same plane. Supplementary Figs. 2 and 4 show Co always displays a relative higher electron density within the center area compared to Fe. Moreover, we simulated the AFM images of fully planar MPcs on a Cu substrate (no structural relaxations performed) as a baseline (Supplementary Fig. 1b, d). These two additional images confirm that the characteristic features of Fe and Co within MPcs are not derived from their relative adsorption heights, but from their different orbital occupations. The relatively elongated axial ligand of Fe’s pushes its ${d}_{{xy}}$ orbital toward the Fermi level. To visualize the nature of the orbitals that contribute to the AFM images and force spectra, we combine DFT one-electron wavefunctions of the states that are within an energy window of 2.49 eV below the Fermi level, in which major peaks of FePc’s spin down states are included. Since it is unclear to what extent the electronic states can contribute to the measured AFM signal, we tested other cutoff energies. We find that: (1) when fewer states (<1.00 eV) are included, no clear features are obtained; (2) when more states (>4.00 eV) are included, the orbital features become less distinct (see Supplementary Fig. 3 for details). We find the major contribution from Fe is ${d}_{{xy}}$ whereas it is not obvious which component(s) are dominant for Co. To obtain a better representation of the wavefunctions, we plot 2D volume slices across the metal atoms (Fig. 4c). For Fe, in the x–y plane, the ${d}_{{xy}}$ component clearly dominates, while no apparent orbital signature is observed in the x–z and y–z planes. For Co, ${d}_{{z}^{2}}$, ${d}_{{xz}}$ and ${d}_{{yz}}$ show a strong intensity in the x–z and y–z planes, consistent with the calculated PDOS. More importantly, these findings qualitatively explain the features observed on the AFM images and force spectra. As for the Fe atom, the larger ${d}_{{xy}}$ contribution makes it appear like a square since ${d}_{{xy}}$ lies between *x* and *y* axes that are along the directions of the N-Fe-N bonds. In contrast, the lack of *z* components in Fe results in a relatively darker spot (smaller repulsive force). For the Co atom, large contributions from ${d}_{{z}^{2}}$, ${d}_{{xz}}$ and ${d}_{{yz}}$ results in a relatively brighter spot (larger repulsive force). The four distinct lobe features (along the Co-N bonds) can be ascribed to the relatively small contribution of ${d}_{{xy}}$. Our results suggest that those occupied states with distinct signatures near the Fermi level play a key role in terms of AFM imaging and force measurement. This analysis provides a rationale for the observed shape difference of Fe and Co atoms within phthalocyanine molecules.

## Conclusion

Recent developments in AFM have provided images of organic molecules on surfaces with remarkable atomic resolution. However, details of the imaging mechanism are still unclear. In particular, one important question concerns the role of the electron density in the measured images; specifically, do the images involve the contributions of all occupied electronic states? Or are they determined only by the states within a relatively small energy interval below the Fermi energy, which are characterized by a slower decay of the wavefunctions above the surface? We selected FePc and CoPc as a stringent model to test AFM capability to distinguish atoms differing by only one atomic number. We found that the Fe and Co centers can be distinguished using both AFM imaging and force spectroscopy. Our DFT calculations further reveal that the differences observed in HR-AFM images originate from the different occupations of the out-of-plane $3{d}$ orbitals of the Fe and Co atoms. These distinct occupations can explain the 5-pN offset measured in the force spectra. Our results show that the states near the Fermi level, rather than the entire electron density, have the largest impact on the AFM images and force spectra, since the wavefunctions of deeper states decay faster and thus have less contribution to the orbital signatures. These results also demonstrate that direct observation of electron orbital signatures is a promising approach to distinguish different atoms within molecules, with potential applications in identifying chemically active sites and for elucidating the catalytic mechanism of MPc-based reactions, such as O_2_^33^ and CO_2_^34^ reduction.

## Methods

### Experimental parameters

Our experiments were performed with a commercial low-temperature combined STM/AFM system (CreaTec) under ultrahigh vacuum and a temperature of ~5 K. The qPlus sensor has a resonance frequency of 33 KHz with a spring constant *k* = 1800 N/m. After gluing on a Pt/Ir tip, the resonance frequency dropped to 31 KHz. In our measurement, the quality factor is about 20,000. To minimize the crosstalk between the qPlus signal and the STM channel, no voltage was applied to the tip during the force measurement process. We set the oscillation amplitude to be 100 pm. The FePc/CoPc molecules were evaporated in two steps from a silicon chip through a direct heating method and deposited on the substrate. FePc was deposited and imaged first, then CoPc was deposited on the surface and examined. By directly comparing images of CoPc with FePc, the difference between them can be distinguished. The Cu substrate was kept at 5 K during the entire experiment.

### Image analysis

For the apparent length measurement based on AFM images, such as the calculations performed in Fig. 2d and the inset of Fig. 3d, we converted the image in RGB scale into a 2D numerical array with values of *I*, which corresponds to the luminance signal, based on a standard weighted sum of the R, G and B components^35^:

1 $$I=0.2989{{{{{\rm{R}}}}}}+0.5870{{{{{\rm{G}}}}}}+0.1140{{{{{\rm{B}}}}}}$$

### DFT modeling and computations

We employed a real-space pseudopotential DFT code—PARSEC^36–38^ for all the calculations. We assume the electron wavefunctions vanish outside a spherical (for free-standing molecules) or a slab (for molecule-on-surface systems) domain. The boundary sphere radius for each system is chosen to be sufficiently large so that we can obtain converged results. We use the extended limited memory Broyden-Fletcher-Goldfarb-Shanno algorithm^39^ for structural relaxation calculations. We set the grid spacing to be 15.9 pm. The density-weighted self-consistent residual error (SRE) was less than 10^−4^ Ry. We modeled the substrate by a 4-layer 8 × 10 Cu(111) surface with the bottom two layers fixed during relaxation. For FePc on Cu(111), we directly used the relaxed structure from our previous work^24^. For CoPc on Cu(111), we started with the same adsorption geometry as optimized for the FePc molecule and then performed structural relaxations. See Supplementary Information for more details (AFM simulation methods, pseudopotentials and exchange-correlation functionals).

We computed the vertical interaction forces as a function of tip height (Fig. 3b) by placing the tip on top of the metal atoms. Here, the CO tip, the MPc molecule and the Cu(111) substrate were all included in these calculations. We employed a four-point central finite-difference formula with a step size of 15.9 pm for these force calculations based on the computed total energies. For the calculation of the 3D distribution of interaction forces (Fig. 3d), we directly took the negative of the first derivative of the calculated total tip-sample energy map using a two-point central finite-difference formula.

The local magnetic moments, ${\mu }_{j}^{{{{{{{\rm{local}}}}}}}}$, of the metal atoms are calculated within spherical domains, ${\Omega }_{j}$^40^:

2 $${\mu }_{j}^{{{{{{{\rm{local}}}}}}}}={\int }_{{\Omega }_{j}}\left[{\rho }_{\uparrow }\left(\vec{r}\right)-{\rho }_{\downarrow }\left(\vec{r}\right)\right]{d}^{3}\vec{r}$$

where ${\rho }_{\uparrow }$ and ${\rho }_{\downarrow }$ are the electronic densities of majority and minority spin.

## Supplementary information

Supplementary information

## Data Availability

The data supporting our results can be found within this article and the Supplementary Information. The Supplementary Information contains details of our AFM image simulation method, more experimental/simulated AFM images, sample electron density maps at different tip heights, and another set of experimental results.
