# Supplementary material for: Observation of electron orbital signatures of single atoms within metal-phthalocyanines using atomic force microscopy
Source: Nat Commun. 2023 Mar 16;14:1460. doi: 10.1038/s41467-023-37023-9 (PMC10020477; doi:10.1038/s41467-023-37023-9)
Supplement: Supplementary file 1 — Supplementary information [file 41467_2023_37023_MOESM1_ESM.pdf]

Supplementary Information

Observation of Electron Orbital Signatures of Single Atoms within Metal-Phthalocyanines using Atomic Force Microscopy

Authors: Pengcheng Chen,[1,†] Dingxin Fan,[1,2,†] Annabella Selloni,[3] Emily A. Carter,[4,5] Craig B. Arnold,[1,4] Yunlong Zhang,[6] Adam S. Gross,[6] James R. Chelikowsky,[2,7,8,*] and Nan Yao[1,*]

Affiliations:

1, Princeton Materials Institute, Princeton University, Princeton, New Jersey, 08540-8211, USA

2, McKetta Department of Chemical Engineering, University of Texas at Austin, Austin, Texas 78712-1589, USA

3, Department of Chemistry, Princeton University, Princeton, New Jersey, 08544-0001, USA

4, Department of Mechanical and Aerospace Engineering and the Andlinger Center for Energy and the Environment, Princeton University, Princeton, New Jersey, 08544-5263, USA

5, Princeton Plasma Physics Laboratory, Princeton, New Jersey, 08540-6655, USA

6, ExxonMobil Technology and Engineering Company, Annandale, New Jersey, 08801-3096, USA

7, Department of Physics, University of Texas at Austin, Austin, TX 78712-1192, USA

8, Center for Computational Materials, Oden Institute for Computational Engineering and Sciences, University of Texas at Austin, Austin, TX 78712-1229, USA

[†] These authors contribute equally

* To whom correspondence should be addressed: E-mail: nyao@princeton.edu; jrc@utexas.edu

Supplementary Methods

Additional Simulation Methods

For AFM image simulations, we employ a quantum based computational method developed by J. R. Chelikowsky and coworkers[1], which differs from the particle-probe model[2] (a charge-density-based method). Although some groups[3, 4, 5] model the tip with additional Cu atoms above the CO molecule, we find the inclusion of a Cu cluster has a negligible effect in terms of the interaction energy[6, 7, 8, 9]. As a result, we model the CO tip with a previously optimized CO molecule. We generate 2D raster grids in the x-y plane over the sample molecules at three different tip heights (separated by 15.9 pm) and compute the total energy of the tip-sample system (E_{ts}) at each grid point. We then combine the small amplitude approximation and the three-point central finite-difference method to compute the relative frequency shift (Δf)[1, 10]. The tip height in simulation is defined as the distance above the averaged z-coordinate of each MPc molecule (we set 312 pm to be our zero point). In addition, we use a tip-tilting correction developed by Guo *et al.*[11] to account for the effect of lateral force. A tip-tilting correction, in general, results in more distinct and sharper bond features[5, 12]. We compute the displacement of the CO tip in the x and y directions, $\vec{\Delta}_{lat}(x, y)$, by assuming a linear relationship between the lateral force, $\vec{F}_{lat}(x, y)$, and the lateral displacement using Eq. (1):

$$\vec{\Delta}_{lat}(x, y) = \frac{\vec{F}_{lat}(x, y)}{k_{CO}} \quad (1)$$

where k_{CO} is the lateral spring constant of the CO tip. k_{CO} is an adjustable empirical parameter which is set to be 0.8 N/m here.

Since we have shown that the attraction between the center Fe atom in FePc and the two bridge Cu atoms is not negligible[6], we simulate the AFM images of MPcs with (Supplementary Figs. 1 a&c) and without (Fig. 2c of the main text) Cu substrates. When the substrate is not included, we perform both spin-polarized and spin-paired calculations. In this case, a modification of the substrate could result in a change in the local spin state of the center metal atom. However, when the substrate is included, we only perform spin-polarized calculations because it is not realistic to quench the spin with the presence of the Cu(111) substrate. We find the inclusion of the Cu(111) substrate results in a slightly bright metal center (for both Fe and Co). We attribute this to the trans effect[13]. The interactions

between the metal center in MPcs and the Cu substrate weaken the constraints of the electrons from the nuclei in z direction. The inclusion of the Cu(111) substrate makes the AFM simulations extremely expensive. To reduce the computational cost to a reasonable value, we employ the frozen density embedding theory (FDET)[9, 14] which divides the total charge density of a system ($n^{tot}(\boldsymbol{r})$) into two subsystems – tip ($n^{t}(\boldsymbol{r})$) and the sample ($n^{s}(\boldsymbol{r})$):

$$n^{tot}(\boldsymbol{r}) = n^{t}(\boldsymbol{r}) + n^{s}(\boldsymbol{r}) \tag{2}$$

Then the total energy functional, $E_{tot}[n^{t}(\boldsymbol{r}), n^{s}(\boldsymbol{r})]$, becomes:

$$\begin{aligned} E_{tot}[n^{t}(\boldsymbol{r}), n^{s}(\boldsymbol{r})] &= \iint \frac{\{n^{t}(\boldsymbol{r}) + n^{s}(\boldsymbol{r})\}\{n^{t}(\boldsymbol{r}') + n^{s}(\boldsymbol{r}')\}}{2|\boldsymbol{r}-\boldsymbol{r}'|} d\boldsymbol{r}d\boldsymbol{r}' \\ &+ \int \{V_{nuc}^{t}(\boldsymbol{r}) + V_{nuc}^{s}(\boldsymbol{r})\}\{n^{t}(\boldsymbol{r}) + n^{s}(\boldsymbol{r})\} d\boldsymbol{r} \\ &+ T_{s}[n^{t}] + T_{s}[n^{s}] + T_{s}^{nadd}[n^{t}, n^{s}] + E_{xc}[n^{t} + n^{s}] + E_{nuc} \end{aligned} \tag{3}$$

where V_{nuc}, T_s, E_{xc} and E_{nuc} represent the nuclear potential (or ionic potential when pseudopotentials are used), kinetic energy functional, exchange-correlation energy functional, and nuclear-nuclear interaction energy, respectively. The nonadditive kinetic energy term, $T_{s}^{nadd}[n^{t}(\boldsymbol{r}), n^{s}(\boldsymbol{r})]$, is approximated by adopting the analytic form of the kinetic energy functional proposed by Tran and Wesołowski[15]. Within FDET, we treat $V_{nuc}^{s}(\boldsymbol{r})$ and $n^{s}(\boldsymbol{r})$ as constants which are computed previously using one single full DFT run of the sample system. Next, we define the embedding potential of the sample, $V_{emb}(\boldsymbol{r})$, as:

$$\begin{aligned} V_{emb}(\boldsymbol{r}) &= \int \frac{n^{s}(\boldsymbol{r}')}{|\boldsymbol{r}-\boldsymbol{r}'|} d\boldsymbol{r}' + V_{nuc}^{s}(\boldsymbol{r}) + \frac{\delta E_{xc}[n]}{\delta n}\Big|_{n=n^{tot}} \\ &- \frac{\delta E_{xc}[n]}{\delta n}\Big|_{n=n^{t}} + \frac{\delta T_{s}[n]}{\delta n}\Big|_{n=n^{tot}} - \frac{\delta T_{s}[n]}{\delta n}\Big|_{n=n^{t}} \end{aligned} \tag{4}$$

and the ordinary Kohn-Sham potential of the tip as the effective potential, $V_{eff}^{t}(\boldsymbol{r})$:

$$V_{eff}^{t}(\boldsymbol{r}) = \int \frac{n^{t}(\boldsymbol{r}')}{|\boldsymbol{r}-\boldsymbol{r}'|} d\boldsymbol{r}' + V_{nuc}^{t}(\boldsymbol{r}) + \frac{\delta E_{xc}[n]}{\delta n}\Big|_{n=n^{t}} \tag{5}$$

We can then obtain the following Schrödinger-like equation in FDET using Eqs. (4) and (5):

$$\left[\frac{-\nabla^{2}}{2} + V_{eff}^{t}(\boldsymbol{r}) + V_{emb}(\boldsymbol{r})\right]\psi_{i}^{t}(\boldsymbol{r}) = \epsilon_{i}^{t}\psi_{i}^{t}(\boldsymbol{r}) \tag{6}$$

where ϵ_{i}^{t} and ψ_{i}^{t} are a set of Kohn-Sham eigenvalues and wavefunctions.

Pseudopotentials

We use Troullier-Martin norm-conserving pseudopotentials[16]. Specifically, for Fe, Co and Cu atoms, we use relativistic spin-polarized calculations to construct their pseudopotentials. Although other construction methods have been proposed for dealing with transition-metal atoms[17, 18, 19], we did not employ the methods in this work as our pseudopotentials (for systems like FePc/CO-FePc with and without Cu(111) substrates) provided good results in our previous work[6]. In addition, we find that including a nonlinear core correction[17] for Fe and Co (tested isolated FePc and CoPc molecules only) does not induce any influential structural change or charge redistribution.

Exchange-Correlation Functionals

For the exchange-correlation (EX) functional, we tested local density approximations by Ceperley-Alder (CA)[20] and by Perdew-Wang (PW92)[21]. As CA and PW92 give similar results (adsorption height, molecular geometry and simulated AFM images), the calculations shown in the main text refer to CA only. A recent theoretical study on FePc and CO-FePc systems confirm that the choice of EX functional has very slight influence in terms of Fe-N bond length for a specific spin state[22].

Supplementary Figures

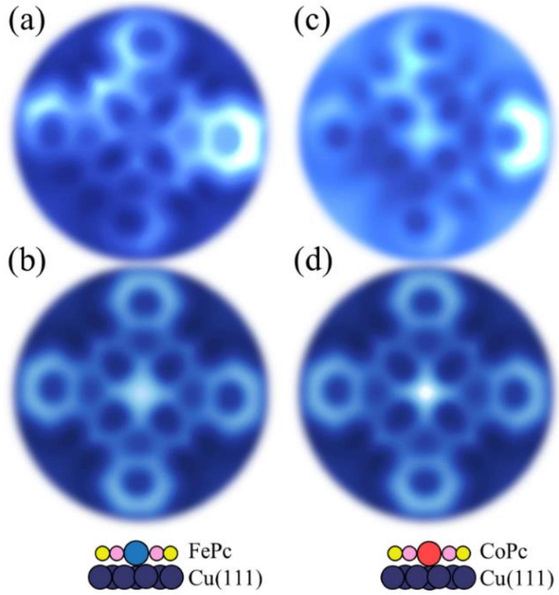

Supplementary Fig 1. Simulated AFM images of FePc and CoPc on Cu(111) at a tip height of -10 pm (CO tip). (a-b) FePc; (c-d) CoPc. The center metal atom is placed at a bridge site. The Cu substrate is included in these spin-polarized DFT calculations. In (b) and (d), the MPc molecules are fully planar (no structural relaxations performed).

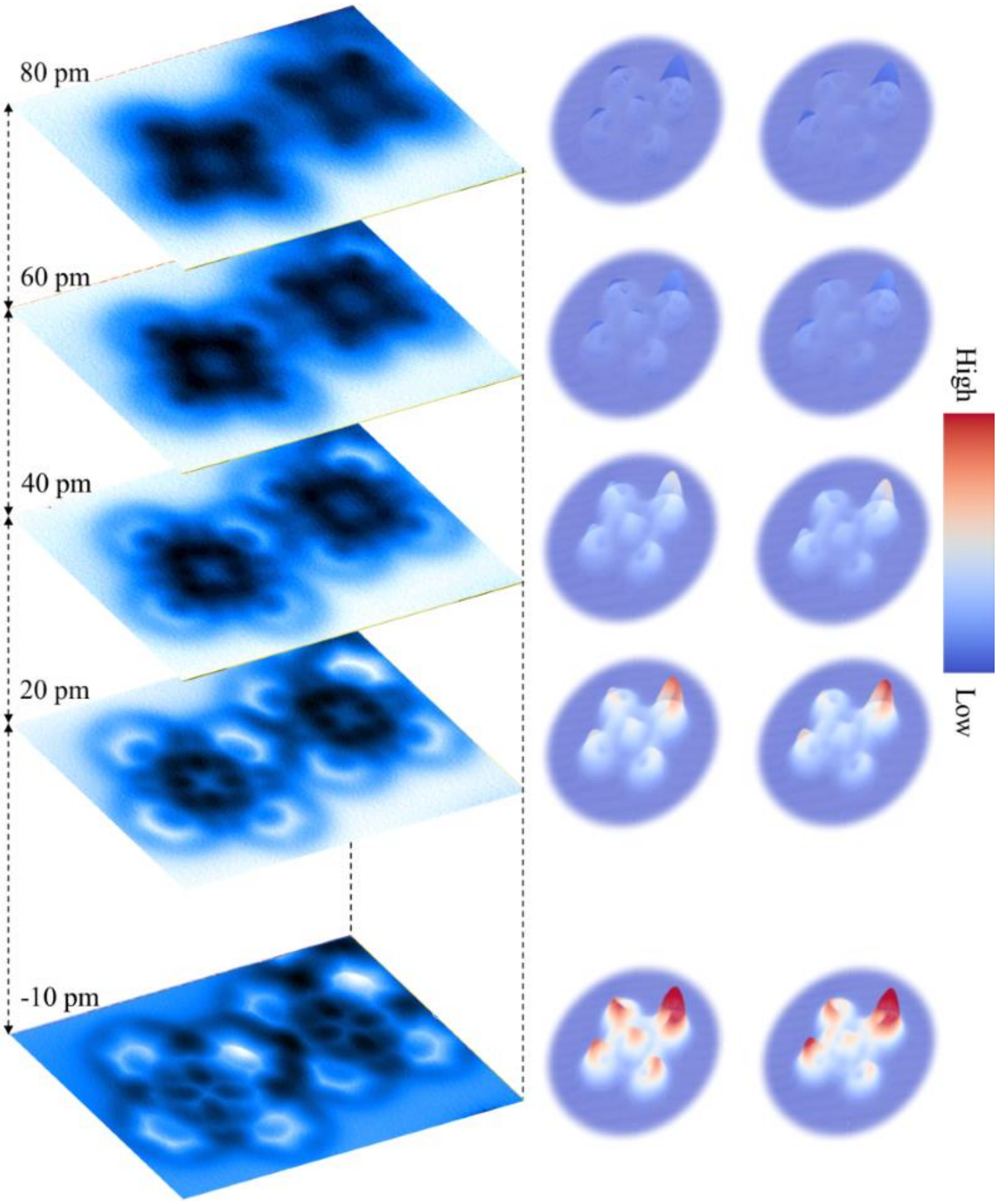

Supplementary Fig 2. Experimental AFM images (left panel) and the corresponding computed 3D electron density maps (right panel) at different tip heights. The experimental tip height is relative to a STM set point (100mV/100pA).

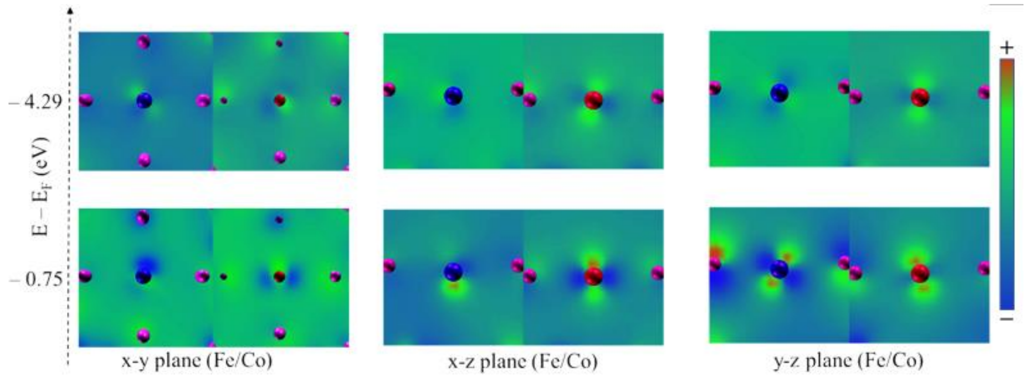

Supplementary Fig 3. 2D volume slice views of combined wavefunctions of MPc on Cu(111). Top panel: states that are 4.29 eV below Fermi level; bottom panel: states that are 0.75 eV below Fermi level. The molecular models are overlapped with the slices. Blue: Fe, red: Co, pink: N atoms.

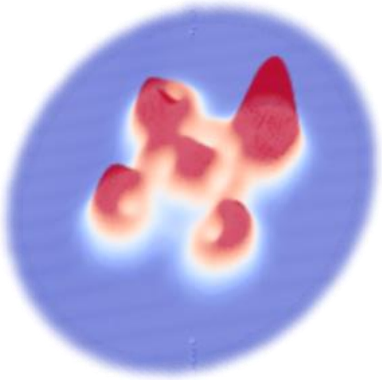

Supplementary Fig 4. 3D electron density maps. The computed 3D electron density map of CoPc when it is intentionally lifted upward by 12.6 pm from the zero point.

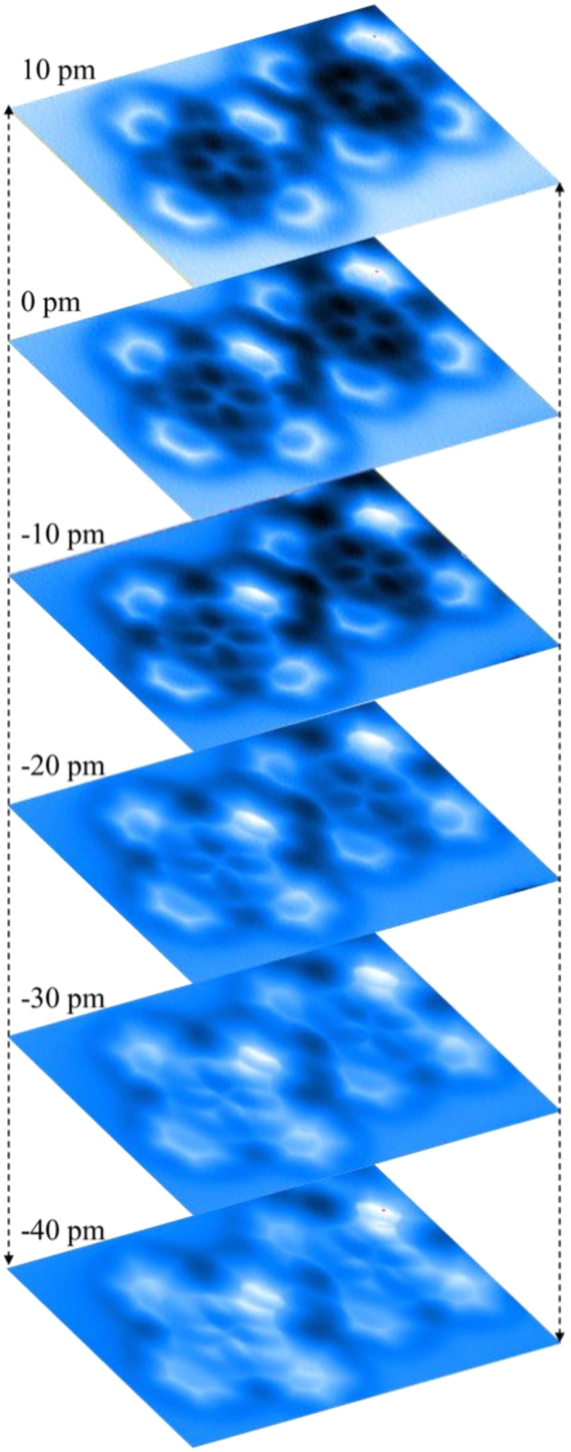

Supplementary Fig 5. Additional experimental AFM images at different tip heights around the zero point. The tip height is relative to a STM set point (100mV/100pA).

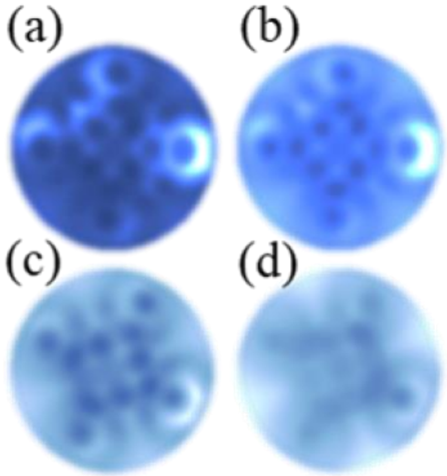

Supplementary Fig 6. Additional simulated AFM images of FePc (a-b) and CoPc (c-d) at larger tip heights. Tip heights are 5.9 pm (a & c) and 21.8 pm (b & d). Spin-polarized DFT calculations are used.

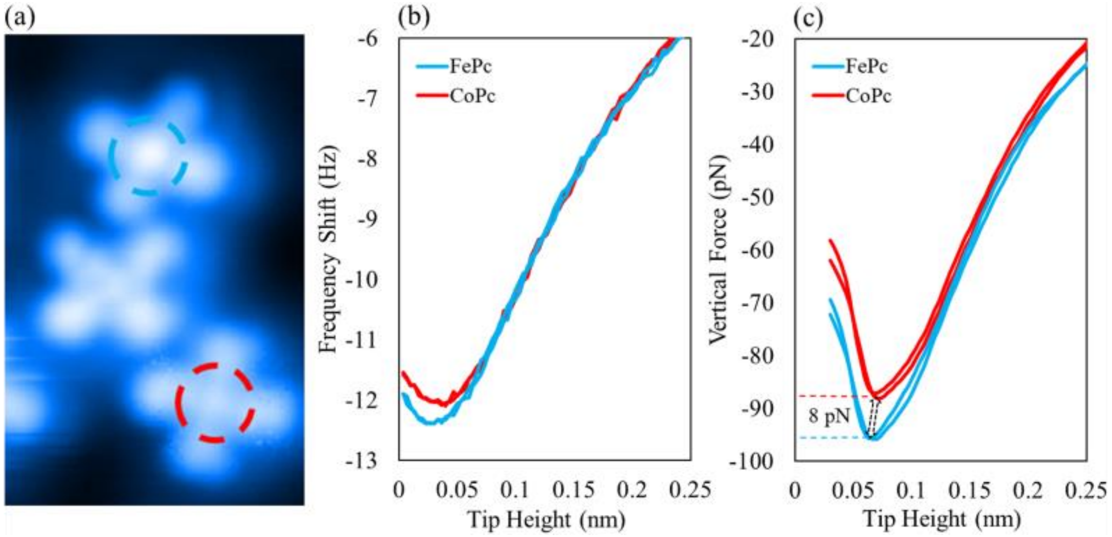

Supplementary Fig 7. Additional experiment performed using a different CO tip show that FePc and CoPc are clearly distinguishable based on either frequency shift or force despite the curves are slightly shifted. (a) STM image of FePc and CoPc molecules using a CO tip (V = 100 mV, I = 30 pA). (b) Measured frequency shift (Hz) and (c) vertical force (pN) acting on the CO tip when it is placed on the top of the (circled in (a)) center metal atom.

Supplementary References

1. Chelikowsky JR, Fan D, Lee AJ, Sakai Y. Simulating noncontact atomic force microscopy images. *Physical Review Materials* **3**, 110302 (2019).

2. Ellner M, Pou P, Pérez R. Molecular Identification, Bond Order Discrimination, and Apparent Intermolecular Features in Atomic Force Microscopy Studied with a Charge Density Based Method. *ACS Nano* **13**, 786-795 (2019).

3. Hapala P, Kichin G, Wagner C, Tautz FS, Temirov R, Jelínek P. Mechanism of high-resolution STM/AFM imaging with functionalized tips. *Physical Review B* **90**, 085421 (2014).

4. Guo C-S, Van Hove MA, Zhang R-Q, Minot C. Prospects for Resolving Chemical Structure by Atomic Force Microscopy: A First-Principles Study. *Langmuir* **26**, 16271-16277 (2010).

5. Gross L, *et al.* Bond-Order Discrimination by Atomic Force Microscopy. *Science* **337**, 1326-1329 (2012).

6. Chen P, *et al.* Breaking a dative bond with mechanical forces. *Nature Communications* **12**, 5635 (2021).

7. Fan D, Chelikowsky JR. Atomic Fingerprinting of Heteroatoms Using Noncontact Atomic Force Microscopy. *Small* **17**, 2102977 (2021).

8. Fan D, Sakai Y, Chelikowsky JR. Real-space pseudopotential calculations for simulating noncontact atomic force microscopy images. *Journal of Vacuum Science & Technology B, Nanotechnology and Microelectronics: Materials, Processing, Measurement, and Phenomena* **36**, 04H102 (2018).

9. Sakai Y, Lee AJ, Chelikowsky JR. First-Principles Atomic Force Microscopy Image Simulations with Density Embedding Theory. *Nano Letters* **16**, 3242-3246 (2016).

10. Giessibl FJ. A direct method to calculate tip–sample forces from frequency shifts in frequency-modulation atomic force microscopy. *Applied Physics Letters* **78**, 123-125 (2001).

11. Guo C-S, Van Hove MA, Ren X, Zhao Y. High-Resolution Model for Noncontact Atomic Force Microscopy with a Flexible Molecule on the Tip Apex. *The Journal of Physical Chemistry C* **119**, 1483-1488 (2015).

12. Fan D, Sakai Y, Chelikowsky JR. Discrimination of Bond Order in Organic Molecules Using Noncontact Atomic Force Microscopy. *Nano Letters* **19**, 5562-5567 (2019).

13. Hieringer W, *et al.* The Surface Trans Effect: Influence of Axial Ligands on the Surface Chemical Bonds of Adsorbed Metalloporphyrins. *Journal of the American Chemical Society* **133**, 6206-6222 (2011).

14. Wesolowski TA, Shedge S, Zhou X. Frozen-Density Embedding Strategy for Multilevel Simulations of Electronic Structure. *Chemical Reviews* **115**, 5891-5928 (2015).

15. Tran F, Wesołowski TA. Link between the kinetic- and exchange-energy functionals in the generalized gradient approximation. *International Journal of Quantum Chemistry* **89**, 441-446 (2002).

16. Troullier N, Martins JL. Efficient pseudopotentials for plane-wave calculations. *Physical Review B* **43**, 1993-2006 (1991).

17. Louie SG, Froyen S, Cohen ML. Nonlinear ionic pseudopotentials in spin-density-functional calculations. *Physical Review B* **26**, 1738-1742 (1982).

18. Watson SC, Carter EA. Spin-dependent pseudopotentials. *Physical Review B* **58**, R13309-R13313 (1998).

19. Cocula V, Pickard CJ, Carter EA. Ultrasoft spin-dependent pseudopotentials. *The Journal of Chemical Physics* **123**, 214101 (2005).

20. Ceperley DM, Alder BJ. Ground State of the Electron Gas by a Stochastic Method. *Physical Review Letters* **45**, 566-569 (1980).

21. Perdew JP, Wang Y. Accurate and simple analytic representation of the electron-gas correlation energy. *Physical Review B* **45**, 13244-13249 (1992).

22. Pichierri F. Theoretical insights into the nature of the bonding between carbon monoxide and iron(II) phthalocyanine: How do QTAIM descriptors change as a function of the Fe–CO distance? *Chemical Physics Letters* **804**, 139901 (2022).
